# Histone Acetyltransferase KAT2A Stabilizes Pluripotency with Control of Transcriptional Heterogeneity

**DOI:** 10.1002/stem.2919

**Published:** 2018-10-17

**Authors:** Naomi Moris, Shlomit Edri, Denis Seyres, Rashmi Kulkarni, Ana Filipa Domingues, Tina Balayo, Mattia Frontini, Cristina Pina

**Affiliations:** ^1^ Department of Genetics University of Cambridge Cambridge United Kingdom; ^2^ Department of Haematology University of Cambridge Cambridge United Kingdom; ^3^ National Health Service Blood and Transplant University of Cambridge Cambridge United Kingdom; ^4^ NIHR BioResource‐Rare Diseases University of Cambridge Cambridge United Kingdom; ^5^ BHF Centre of Excellence, Division of Cardiovascular Medicine Addenbrooke's Hospital, University of Cambridge Cambridge United Kingdom

**Keywords:** Embryonic stem cells, Epigenetics, Gene expression pluripotency

## Abstract

Cell fate transitions in mammalian stem cell systems have often been associated with transcriptional heterogeneity; however, existing data have failed to establish a functional or mechanistic link between the two phenomena. Experiments in unicellular organisms support the notion that transcriptional heterogeneity can be used to facilitate adaptability to environmental changes and have identified conserved chromatin‐associated factors that modulate levels of transcriptional noise. Herein, we show destabilization of pluripotency‐associated gene regulatory networks through increased transcriptional heterogeneity of mouse embryonic stem cells in which paradigmatic histone acetyl‐transferase, and candidate noise modulator, Kat2a (yeast orthologue Gcn5), have been inhibited. Functionally, network destabilization associates with reduced pluripotency and accelerated mesendodermal differentiation, with increased probability of transitions into lineage commitment. Thus, we show evidence of a relationship between transcriptional heterogeneity and cell fate transitions through manipulation of the histone acetylation landscape of mouse embryonic stem cells, suggesting a general principle that could be exploited in other normal and malignant stem cell fate transitions. stem cells *2018;36:1828–11*

**Significance Statement**

Pluripotent mouse embryonic stem cells (mESCs) are capable of differentiating toward all mature cell types through cell fate transition events, which are often associated with increased transcriptional heterogeneity. Yet, there is little understanding of the molecular mechanisms underlying this phenomenon. Through inhibition of the histone acetyltransferase Kat2a in pluripotent mESCs, an increase in transcriptional heterogeneity (as measured by coefficient of variation) and an increased probability of exit from pluripotency is observed. The results of this study suggest the existence of an important relationship between chromatin modification, transcriptional heterogeneity, and cell fate decisions, which is likely to be important in understanding development and disease.

## Introduction

Cellular states, and their associated transitions, are a function of the transcriptional programs active in the cell, which in turn depend on the chromatin configuration of their respective genes. It has been observed in multiple mammalian differentiation and developmental systems, including in mouse embryonic stem (ES) cells, that the cellular states during transition events are heterogeneous at the transcriptional level 1, 2, 3, 4, 5, 6, 7. Such heterogeneity is thought to reflect temporal variability in gene expression, within individual cells, in an uncoordinated manner 8. When variability affects genes that are regulators or effectors of cell fates, the expression status of individual genes can endow cells with different probabilities of effecting a transition 9, eventually resulting in a proportion of cells acquiring the new fate; thus, heterogeneity in expression of these genes would vary the transition probability from a given state.

The homeobox gene *Nanog* is a paradigmatic pluripotency regulator that exhibits such variability in gene expression 10, 11, 12. *Nanog* is strictly required for establishment of pluripotency, both in vitro and in the embryo 13, but is dispensable for its maintenance 11. *Nanog* transcriptional reporters have been used to prospectively isolate cells on the basis of expression levels and, while there is some reversibility between Nanog high and low expression states, Nanog low cells have a higher probability of exiting self‐renewal into differentiation 10, 12. A role of Nanog down‐regulation in the probabilistic exit from pluripotency is supported by experiments coupling reversible Nanog knockdown with single‐cell transcriptomics showing that remodeling of pluripotency networks associated with Nanog loss can be transiently reversed 14.

The Nanog transcriptional reporters that are based on stable green fluorescent protein (GFP; heterozygous TNGA cells) 11 exhibit a trimodal distribution of high, mid and low GFP populations. While the high and low states represent the active and inactive transcriptional state of Nanog, respectively, the mid‐Nanog (MN) population is likely to contain cells in which the Nanog promoter has been recently switched off, reversibly or irreversibly, causing the GFP levels to decay. This population is less apparent in destabilized fluorescent reporters such as the destabilized Venus reporter line, Nanog‐venus‐nuclear localization signal‐pEST degradation signal (VNP) 15, confirming that intermediate levels of expression are not sustainable and resolve rapidly into high (HN) or low (LN) states. Therefore, in theory, the MN population should encompass all bona fide early transition events out of pluripotency and into lineage commitment. However, its transient nature makes it difficult to probe the molecular programs of the state transition separate from protracted GFP expression, or confounding dissociation between reporter and endogenous Nanog expression 16.

Assessing the mechanistic basis of the transition out of pluripotency can be finely achieved through the use of Nanog reporter systems and it may shed light on a putative contribution of transcriptional heterogeneity to the probabilistic nature of cell state transitions. Dynamic changes in transcriptional activity, and the resulting changes in state‐transition probabilities, are likely to be regulated, at least in part, at the level of histone lysine acetylation. In yeast, amplitude and frequency of transcriptional bursting 17 are regulated by distinct histone acetyl‐transferase and deacetylase complexes which determine levels of H3K9 acetylation (H3K9ac) in the promoter and the body of the gene 18. Promoter acetylation influences transcriptional variability or noise, as measured by coefficient of variation (CV = standard deviation/mean). Loss of the histone acetyl‐transferase *Gcn5* or its partner *Sgf29* result in increased noise, while loss of components of the Rdp3s histone deacetylase (HDAC) complex, which increase levels of H3K9ac, reduce gene expression CV.

To achieve a global reduction of H3K9ac in mouse ES cells, we chemically inhibited Gcn5 with the (2R,3S)‐rel‐4‐Methylene‐5‐oxo‐2‐propyltetrahydrofuran‐3‐carboxylic acid (MB‐3) compound 19, which we have recently validated to phenocopy loss of Gcn5 homolog KAT2A in mammalian cells 20. While this resulted in minimal changes to average gene expression levels, it caused a significant enhancement of expression heterogeneity for a number of genes, including *Nanog*, with associated remodeling of gene regulatory networks (GRNs). These changes associated with functional destabilization of pluripotency and accelerated differentiation, namely to mesendodermal (ME) lineages. Our results suggest that increased transcriptional heterogeneity may not only reflect but indeed mechanistically promote state transitions in mammalian cells.

## Materials and Methods

### Cell Culture

E14Tg2A, TNGA 11, Nanog‐VNP 15, Sox1‐GFP 21, and T‐GFP 22 mouse ES cells were maintained or differentiated as described 23. 2i medium used N2B27 (NDiff 227, Takara, Saint‐Germain‐en‐Laye, France) with 1 μM PDO325901 (R&D, Abingdon, UK) and 3 μM Chiron (Cambridge Wellcome Trust/Medical Research Council Stem Cell Institute). Kat2a inhibition used 100 μM MB‐3 (ab141255, AbCam, Cambridge, UK) 19, or an equal volume of dimethyl sulfoxide (DMSO). ES cell colony‐forming capacity was quantified using Alkaline Phosphatase detection kit (Sigma Aldrich, Gillingham, UK) as per manufacturer's instructions.

### Flow Cytometry

Cell sorting was done on MoFlo (Beckman Coulter, High Wycombe, UK), FACSAria III (BD, Wokingham, UK), or Influx (BD) machines, and analysis performed on a BD LSR‐Fortessa, with 4′,6‐diamidino‐2‐phenylindole (DAPI) as a dead cell marker. Apoptosis analysis used Annexin‐V antibody (a13202 Fluor‐568, Invitrogen, Loughborough, UK) and DAPI as per manufacturers' instructions. Cell cycle analysis used Propidium Iodide staining of ethanol‐fixed cells.

### Wash‐Off Experimental Protocol

TNGA cells in serum and leukemia inhibitory factor, LIF (ESLIF) were grown for 1 or 2 days with DMSO or MB‐3 (50 and 100 μM), or exposed to control N2B27 pro‐differentiation conditions. At the end of the treatment, cells were washed of the treatment, their GFP profile assessed, and cultured for up to 5 days in ESLIF with daily monitoring of distribution of GFP levels (Supporting Information Fig. S3A). The composite GFP distribution obtained from randomly sampled cells from all time‐points and conditions was best described by three Gaussian curves that correspond to the HN, MN, and LN states 24 (Supporting Information Fig. S3B), and every cell was assigned to one of these states using a probabilistic soft clustering approach (see Supporting Information Data). Population proportions were calculated for each condition at each time point, and kinetic modeling used to estimate rates of transition between states and general state reversibility (Fig. 3B, 3C).

### RNA Sequencing

RNA extracted from TNGA cells treated in triplicate for 48 hours in ESLIF with DMSO or MB‐3 (100 μM), was used for polyA library preparation and sequencing on an Illumina HiSeq4000 instrument (Illumina, Cambridge, UK). Details of alignment, quantification, differential gene expression and data analysis are described in Supporting Information Data. Data have been deposited in GEO (GSE114797).

### Chromatin Immunoprecipitation Sequencing

Chromatin was prepared from TNGA cells treated in duplicate for 48 hours in ESLIF with DMSO or MB‐3 (100 μM), and immunoprecipitated with an anti‐H3K9ac antibody (ab4441, AbCam), as described 20. Details of alignment, peak calling and identification, and data analysis are described in detail in Supporting Information Data. Data have been deposited in GEO (GSE114797).

### Single‐Cell Quantitative Reverse Transcription Polymerase Chain Reaction

Single‐cell quantitative reverse transcription polymerase chain reaction (scRT‐qPCR) followed the Fluidigm Two‐Step Single‐Cell Gene Expression method on a 96.96 Dynamic Array IFC and the BioMark HD system (Fluidigm, London, UK), with EvaGreen Supermix 25 (primers in Supporting Information Table S1) or Taqman Assays (Supporting Information Table S2). Commercially available spike‐ins (Fluidigm, C1 RNA Standards Assay Kit, PN100‐5582) controlled for technical variability. Further details of analysis are described in Supporting Information Data.

## Results

### Kat2a Inhibition in Mouse ES Cells Decreases H3K9ac and Enhances Heterogeneity of Nanog Expression

**Figure 1:**
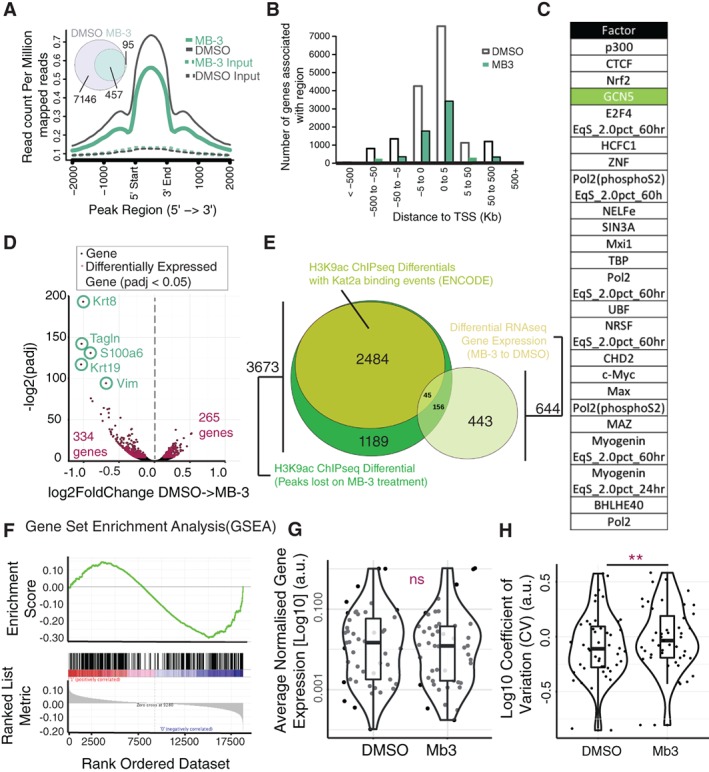
Effects of Kat2a inhibition on H3K9ac and transcriptional heterogeneity of mouse embryonic stem (ES) cells. **(A):** MB‐3 treatment of TNGA mouse ES cells results in specific loss of H3K9ac. Height and number (DMSO: 11724; MB‐3: 4673, 98% overlap) of H3K9ac peaks are significantly reduced after 2 days of MB‐3 treatment. **(B):** H3K9ac marks associated with TSS maintain their location profiles following MB‐3 treatment but reduce in number. **(C):** Transcription factor binding sites associated with genes that display reduced H3K9ac following MB‐3 treatment include GCN5/Kat2a. List represents transcription factors with Q‐value equal to 0, ordered by proportion of observed H3K9ac differential genes to total number of binding site‐associated genes. **(D):** RNA‐seq analysis of TNGA mouse ES cells treated with MB‐3 for 2 days. Volcano plot highlighting differentially expressed genes (red dots: adjusted *p*‐value <.05). **(E):** Differentially expressed genes partially overlap with H3K9ac gene‐associated peaks lost on MB‐3 treatment. **(F):** Gene set enrichment analysis of Kat2a direct targets in mouse ES cells among MB‐3 down‐regulated genes (ES = −0.30, NES = −1.34; *p* value = .005). The negative enrichment score of direct targets (top panel) along the differential RNA‐seq dataset ordered by rank (bottom panel) reveals that it is the down‐regulated genes on MB‐3 treatment that are most enriched for Kat2a direct targets. **(G):** Quantification of mean gene expression from Single‐cell quantitative reverse transcription polymerase chain reaction (scRT‐qPCR) from cells treated with DMSO or MB‐3. There is no significant difference in mean gene expression between these conditions (Student's *t* test, *p* > .05). **(H):** CV of genes following scRT‐qPCR shows increase in CV on MB‐3 treatment (Student's *t* test, *p* < .01). Abbreviations: CV, coefficient of variation; DMSO, dimethyl sulfoxide; GSEA, gene set enrichment analysis; TSS, transcriptional start sites.

We treated TNGA cells with MB‐3 and observed a global reduction of H3K9ac both in number and height (Fig. 1A) of acetylation peaks. As expected for H3K9ac mark, the large majority of peaks were located in the vicinity of transcriptional start sites (TSS) (Fig. 1B), and the regions in which acetylation was lost as a result of MB‐3 treatment were significantly enriched for Kat2a/Gcn5 occupancy as per the ENCODE significance tool (Fig. 1C), supporting specificity of MB‐3 activity. Myc/Max and E2F binding was also enriched at these locations, highlighting their well‐described cooperation with Kat2a 26, 27. The loci specifically affected by Kat2a inhibition (Supporting Information Files S1 and S2) associated with metabolic functions, namely mitochondrial, as well as DNA and RNA metabolism (Supporting Information File S3), suggesting an impact on general, rather than tissue‐specific, functions and putative pervasive activities across multiple cell types. Interestingly, the overall reduction in H3K9ac did not translate into substantial changes in gene expression (Fig. 1D), as RNA sequencing (RNA‐seq) analysis of MB‐3 versus DMSO (vehicle)‐treated TNGA cells revealed 599 differentially expressed genes, with only 3 at a fold change ≥2 (Supporting Information File S4). Differentially expressed genes showed overlap with genes with differential H3K9ac and were significantly enriched in Kat2a binding targets (Fig. 1E), namely those recently described in mouse ES cells 26 (Fig. 1F). Given that the reduction in H3K9ac was not matched by significant changes in gene expression levels, we asked whether treatment might instead result in enhanced cell‐to‐cell heterogeneity of transcription, as described for Gcn5 in yeast.

We selected a representative subset of Kat2a binding targets, using the Hirsch et al. dataset to identify 47 targets of interest, and interrogated 70 individual cells (35 DMSO, 35 MB‐3) for transcript levels and cell‐to‐cell transcriptional variability (measured by CV), using scRT‐qPCR. Similar to the RNA‐seq data, we did not find significant differences in mean expression levels between the two treatments (Fig. 1G). However, for the majority of the individual genes analyzed, as well as at a global level, there was a significant increase in CV (Fig. 1H). The data thus support the hypothesis that inhibition of Kat2a‐dependent H3K9ac results in increased transcriptional heterogeneity in mouse ES cells, similarly to previous observations in *Saccharomyces cerevisiae* (supporting information data of 18).

**Figure 2:**
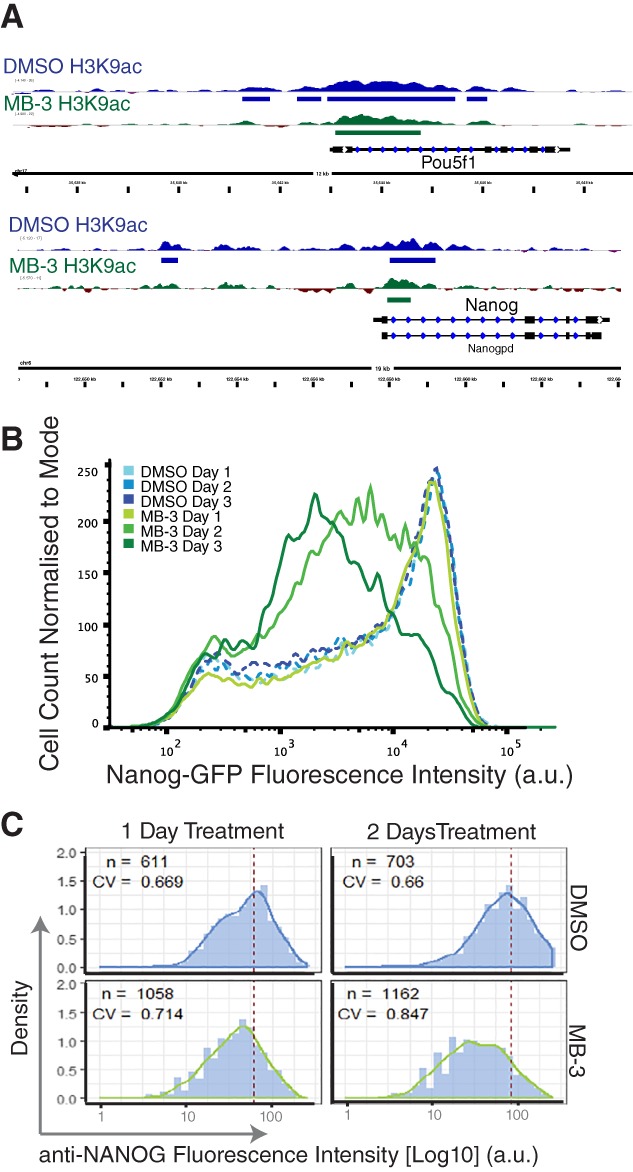
Effect of Kat2a inhibition on Nanog transcriptional heterogeneity. **(A):** Chromatin immunoprecipitation analysis of H3K9 acetylation at the *Pou5f1* (top) and *Nanog* (bottom) loci, showing reduction in height and peak width/number upon MB‐3 treatment. There is specific loss of H3K9ac at the *Pou5f1* promoter immediately upstream of exon E1 included in the pluripotency‐associated Oct4a transcript and protein isoform 29. **(B):** Flow cytometry analysis of heterozygous GFP expression from the *Nanog* locus in TNGA cells upon 1–3 days of MB‐3 treatment; data representative greater than five independent observations. **(C):** Equivalent increase in heterogeneity observed in wildtype E14tg2A cells stained with NANOG antibody (see Supporting Information Methods for quantification details). Abbreviations: CV, coefficient of variation; DMSO, dimethyl sulfoxide; GFP, green fluorescent protein.

We then focused on components of the pluripotency network and asked whether their H3K9ac status was affected by Kat2a inhibition. We found that individual H3K9ac peaks were lost in the proximal upstream regions of the *Pou5f1/Oct4* locus 28 upon MB‐3 treatment (Fig. 2A), an observation that extended to its regulator *Sall4* 30(Supporting Information File S2). Furthermore, we observed a reduction in H3K9ac in the *Nanog* locus (Fig. 2A), including at a distal H3K9ac‐enriched region which might correspond to an enhancer element 31. The impact on Nanog prompted us to check whether Kat2a inhibition with MB‐3‐affected heterogeneity of GFP expression in TNGA cells, which has been associated with altered likelihood of pluripotency and differentiation state transitions 24. Indeed, sustained inhibition of Kat2a activity modified GFP expression from the *Nanog* locus, with increased representation of MN cells and a broader distribution of fluorescence intensity values, distinct from the dominant HN observed in control cells (Fig. 2B). A similar increase in Nanog expression heterogeneity could be reproduced in wild‐type E14tg2A mouse ES cells using immunofluorescence staining of endogenous NANOG protein (Fig. 2C), suggesting that the results were not limited to the TNGA genetic background.

Knockdown of *Kat2a* expression, through lentiviral‐delivered short hairpin RNAs, supports the specificity of inhibitor activity. Kat2a depletion in TNGA cells increases heterogeneity in the distribution of Nanog levels in a manner proportional to the level of Kat2a knockdown, with accumulation of MN and LN cells (Supporting Information Fig. S1A). Similar results are obtained in the Nanog‐VNP cell line 15(Supporting Information Fig. S1B), in which MB‐3 treatment also results in a shift toward lower Nanog levels (Supporting Information Fig. S1C). Overall, the results indicate that loss of Kat2a activity impacts variability of Nanog expression in a manner suggestive of enhanced transition out of pluripotency, as represented by high Nanog levels. We pursued these findings at a functional level to determine the associated impact on cellular state.

### Kat2a Inhibition Impacts Mouse ES Cell Pluripotency and Differentiation

**Figure 3:**
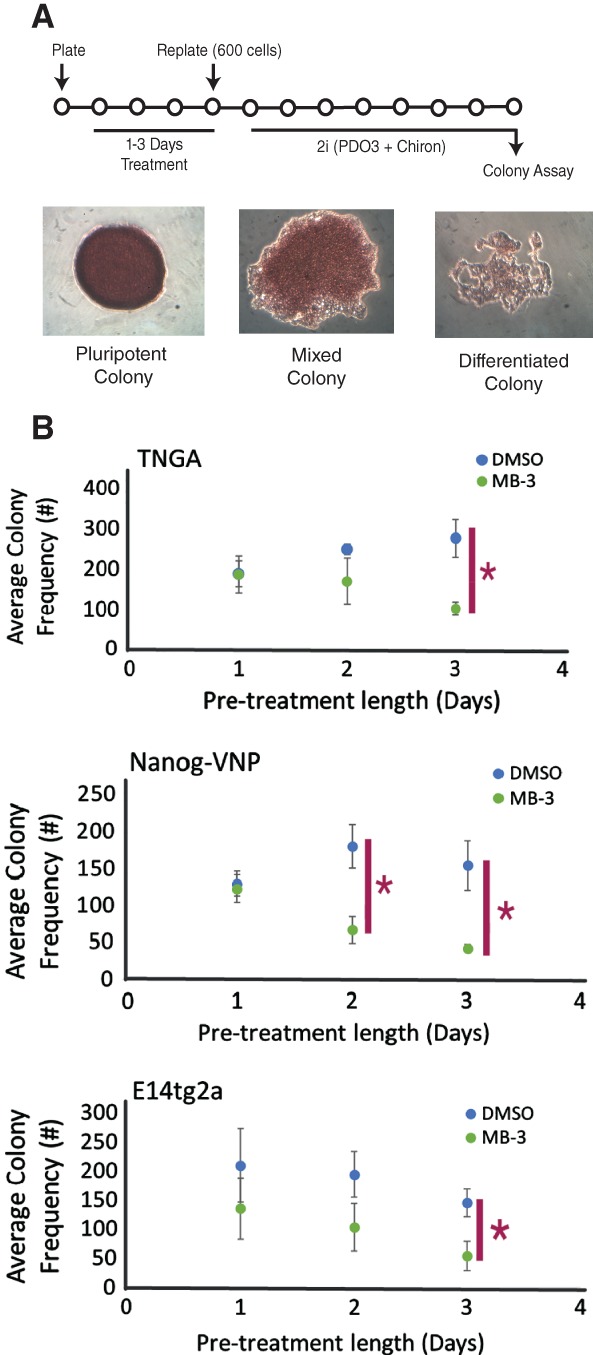
Effects of MB‐3 treatment on naïve pluripotency. **(A):** Experimental design: TNGA cells treated with MB‐3 or DMSO in ESLIF (1–3 days), followed by passaging at clonal density and culture in 2i medium for 7 days. Pluripotency measured by colony number and alkaline phosphatase (AP) staining in colonies, as shown in representative images below. **(B):** Significantly reduced number of AP+ pluripotent colonies from MB‐3‐treated cells after 2i medium switch (n = 3; Student's *t* test, *p* < .05). No significant difference was observed in frequencies of mixed or differentiated colonies (data not shown). Abbreviation: DMSO, dimethyl sulfoxide.

We tested the functional impact of Kat2a inhibition on pluripotency by treating TNGA, E14tg2a, and Nanog‐VNP cells with MB‐3 or DMSO in conventional serum and LIF‐containing culture conditions (ESLIF; Fig. 3A). After 1–3 days of treatment, we washed off the inhibitor, and cultured the cells under stringent naïve pluripotency “2i” conditions 32, after which we quantified the number of undifferentiated alkaline phosphatase‐positive colonies obtained. While DMSO exposure had no effect on colony formation, cells exposed to MB‐3 gradually lost the capacity to establish pluripotent colonies (Fig. 3B), compatibly with an increased probability of exiting the pluripotent state. In agreement with these findings, TNGA cells newly cultured in 2i conditions from ESLIF routine culture, in the presence of MB‐3, show a broader GFP peak (Supporting Information Fig. S2A), which is lost as cells undergo increased apoptosis (Supporting Information Fig. S2B), presumably because of the inability of 2i medium to sustain primed and committed states. Cells cultured in ESLIF, conversely, do not show increased apoptosis (Supporting Information Fig. S2B), and their cell cycle status is also not affected (Supporting Information Fig. S2C). Interestingly, cells adapted to 2i conditions over several passages are unaffected by Kat2a inhibition by 2i + MB‐3 treatment (Supporting Information Fig. S2D), which may reflect a change of epigenetic control, and is not dissimilar to loss of Nanog itself in mouse ES cells.

**Figure 4:**
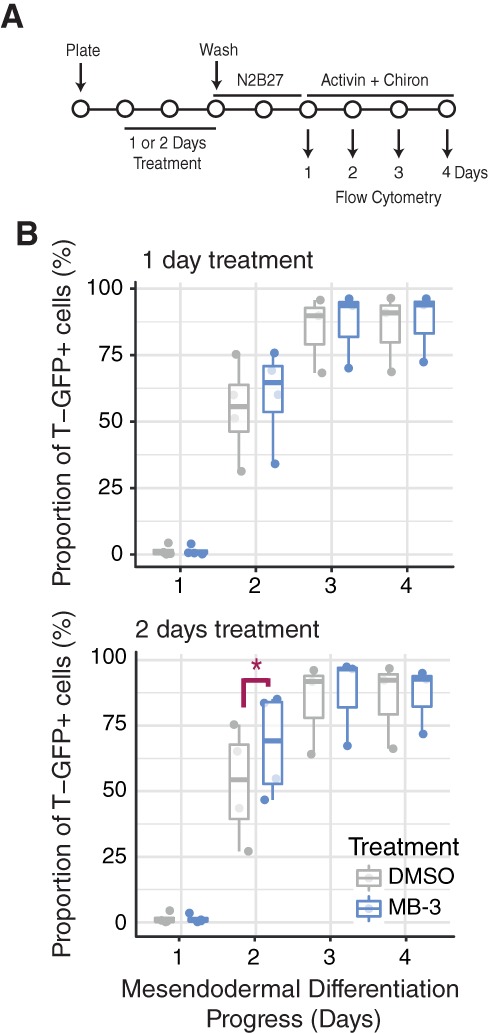
Effect of MB‐3 treatment on mesendodermal differentiation of mouse embryonic stem (ES) cells. **(A):** Mesendodermal differentiation experimental design: Brachyury‐GFP (T‐GFP) cells treated with MB‐3 or DMSO in ESLIF medium (1–2 days) before wash‐off and replating in N2B27 (days 0–1) followed by N2B27 with 100 ng/ml Activin and 3 μM Chiron (days 2–4). Flow cytometry was used to quantify the proportion of GFP+ cells on days 1–4 of differentiation. **(B):** Exposure of mouse ES cells to MB‐3 for 2 days significantly anticipates detection of T‐GFP expression, indicative of accelerated ME commitment (n = 4). Paired Student's *t* test. *p < .05. Abbreviations: DMSO, dimethyl sulfoxide; GFP, green fluorescent protein.

We then asked if Kat2a inhibition promoted differentiation decisions. We took advantage of ES cells with a GFP reporter of ME marker *T* (Brachyury) 22 and treated them for variable periods of time in the presence of DMSO or MB‐3 before washing‐off the treatment and placing the cells under ME‐promoting culture conditions (Fig. 4A). We observed a significant acceleration of T expression in cells exposed to MB‐3 (Fig. 4B). The same, however, was not true of Sox1‐GFP 33 expression in ES cells placed under neuro‐ectodermal (NE) culture conditions after inhibitor treatment (Supporting Information Fig. S2E), suggesting some selectivity in lineage commitment decisions.

### Kat2a Inhibition Captures a MN Transition State in Mouse ES Cells

**Figure 5:**
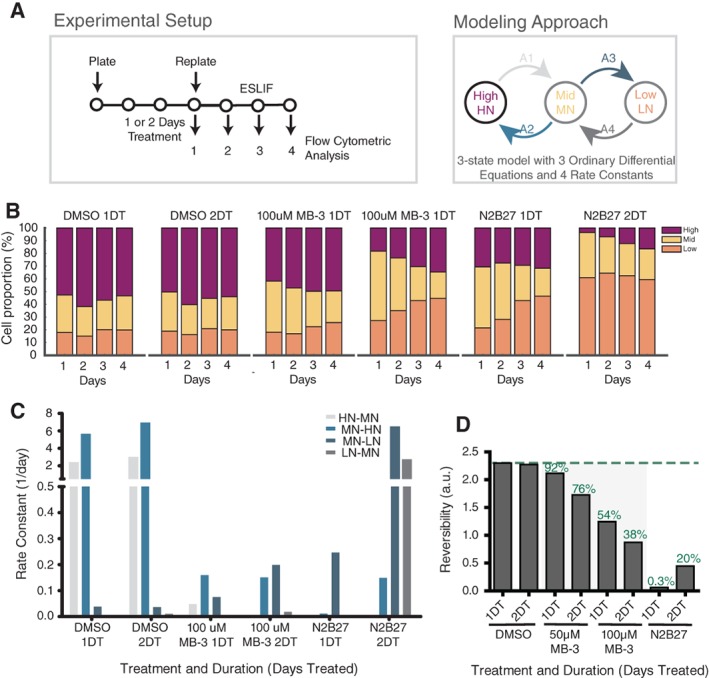
Inhibition of Kat2a catalytic activity affects reversibility of commitment decisions in TNGA mouse embryonic stem cells. **(A):** Experimental design of wash‐off experiments (left) and modeling approach (right). TNGA cells were cultured for 1–2 days in ESLIF medium with MB‐3 (50 or 100 μM) or DMSO, or in N2B27 differentiation medium. Cells were washed off the treatment, and cultured for up to 3 days in ESLIF, with daily monitoring of Nanog‐GFP profile. Four independent biological replicas were performed for accurate model fitting. A model was constructed using the proportion of cells in each of the three HN, MN, and LN states with four kinetic parameters (A1‐4; see Supporting Information Methods). **(B):** Proportion of cells assigned to each state (HN, MN, or LN) on each day of the protocol, per treatment. **(C):** Kinetic modeling of transition rates between HN, MN, and LN states in response to transient exposure to MB‐3 (DMSO or N2B27, controls). HN‐to‐MN represents exit from pluripotency/commitment to differentiate; MN‐to‐LN represents differentiation; reversibility of either decision is given by the reverse transition probability. **(D):** Reversibility indices (MN‐to‐HN + LN‐to‐MN)/(HN‐to‐MN + MN‐to‐LN) for different doses and durations of treatment. Green text indicates equivalent percentage of DMSO treatment. Abbreviations: DMSO, dimethyl sulfoxide; DT, days treatment; GFP, green fluorescent protein; HN, high Nanog‐GFP; MN, mid Nanog‐GFP; LN, low Nanog‐GFP.

The impact of MB‐3 treatment on pluripotency and ME differentiation is suggestive of a change in the probability of state transition, rather than with an absolute requirement to either state. To measure the probability of ES cells moving from pluripotency toward priming and commitment and the reversibility of these transitions, we used the TNGA cells and modeled rates of transition between HN (pluripotent), MN (primed), and LN (committed) states upon treatment exposure and wash‐off (Fig. 5A; see section for details).

DMSO‐treated cells were unchanged by the treatment and transited between HN and MN states with a slight advantage toward the reverse movement, sustaining a dominant HN population. N2B27‐treated cells showed a clear movement toward the LN state, denoting irreversible differentiation, which was dependent on the duration of treatment. MB‐3‐treated cells displayed a unique pattern of long‐term retention in the MN state, with slow transition rates in either direction and a progressive dose and time‐dependent decrease in reversibility (Fig. 5B, 5C and Supporting Information Fig. S3A, S3B). A similar pattern was observed in TNGA sorted as MN and exposed to MB‐3 (Supporting Information Fig. S3C). Overall, the data suggest that inhibition of Kat2a‐mediated acetylation permits the capture of mouse ES cells at the exit of pluripotency; in doing so, it may enrich for an otherwise fleeting state of transition out of pluripotency and into early ME differentiation.

### MB‐3‐Driven Pluripotency‐To‐ME Transition Associates with Transcriptional Heterogeneity

**Figure 6:**
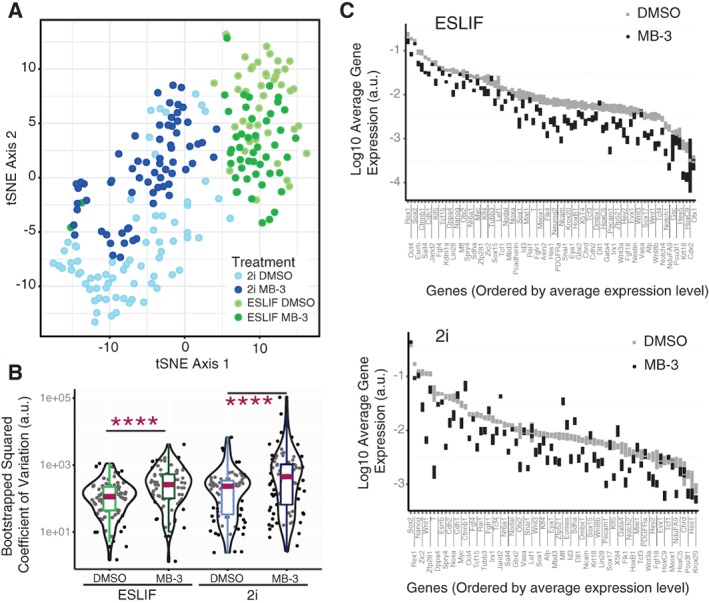
MB‐3 treatment of TNGA mouse embryonic stem cells results in increased transcriptional heterogeneity from key pluripotency and differentiation regulatory loci. **(A):** T‐distributed stochastic neighbor embedding (t‐SNE) plot of transcriptional profiles of individual TNGA cells treated with DMSO or MB‐3 in ESLIF and 2i conditions. **(B):** Overall changes in gene expression squared coefficient of variation between DMSO and MB‐3‐treated cells assayed from ESLIF (left) and 2i (right) culture conditions (paired Student's *t* test, *p* < .0001). **(C):** Ordered representation of gene expression changes between DMSO and MB‐3‐treated TNGA cells in ESLIF (top) and 2i (bottom) conditions. Mean expression represented as dot, standard error of the mean is represented as vertical bars. Abbreviation: DMSO, dimethyl sulfoxide.

We next sought to characterize the transcriptional architecture of the transition state at single‐cell resolution by interrogating individual cells captured from MB‐3 and DMSO‐treated cultures of mouse TNGA ES cells. We used scRT‐qPCR transcriptional profiling and determined the presence, level, and heterogeneity of expression of a selection of 92 genes with roles in ES cell pluripotency and differentiation (Supporting Information Fig. S4A). We excluded LN cells to avoid the confounding effect of irreversibly committed or differentiated cells, and analyzed 90 TNGA cells from ESLIF cultures, which are permissive to priming, and 138 from recently established naïve 2i cultures, which respond to MB‐3 treatment with a broadened peak of GFP fluorescence intensity (Supporting Information Fig. S2A), but do not sustain priming of differentiation programs. Global representation of the transcriptional programs of individual TNGA cells under the different experimental conditions shows an evident separation between the transcriptional spaces occupied by naïve and primed pluripotency. Importantly, it also reveals that the transcriptional space occupied by MB‐3‐treated 2i cells is in closer proximity to primed pluripotency than control (DMSO‐treated) 2i cells (Fig. 6A), suggesting a requirement for Kat2a activity in establishing or maintaining pluripotency. This is consistent with the findings of Dent and colleagues 26, who identified a requirement for Kat2a in coordination with Myc in establishing induced pluripotency upon reprograming of differentiated somatic cells. In contrast, Kat2a active and inactive primed pluripotency transcriptional landscapes are closely overlapping (Fig. 6A), which may partly reflect the exclusion of cells with lower levels of Nanog, and also denote the slow transit of MB‐3 treated cells out of the primed self‐renewal (Supporting Information Fig. S3B).

We inspected the specific changes in single‐cell gene expression patterns imposed by MB‐3 treatment which underlay a misconfiguration of the 2i transcriptional state. We noted that MB‐3 treated cells in naïve pluripotent conditions significantly enhanced cell‐to‐cell variability, or heterogeneity, of gene expression levels relative to control (Fig. 6B and Supporting Information File S5), a finding reminiscent of the role of Kat2a yeast homolog Gcn5 in transcriptional noise 34. A similar significant gain of gene expression variability was also observed in primed cells upon MB‐3 treatment (Fig. 6B and Supporting Information File S5). Increased heterogeneity occurred at all levels of average gene expression (Supporting Information Fig. S4B), and with no significant change to average gene expression levels (Fig. 6C and Supporting Information Fig. S4D). Frequency of gene expression was more dynamic between conditions, with minimal changes in ES‐LIF, and some significant gains and losses in 2i in the presence of MB‐3 (Supporting Information Fig. S4C). We did not observe the presence of new cell clusters within ES‐LIF or 2i cultures upon MB‐3 treatment (Supporting Information Fig. S4E), suggesting that the observed gain in heterogeneity upon Kat2a inhibition results from transcriptional perturbation and does not arise from the de novo emergence of new cell populations. We sought to articulate changes in gene expression frequency and variability of transcript levels through inference of GRNs, to understand the contribution of transcriptional heterogeneity to regulatory programs at the exit of pluripotency.

### MB‐3‐Driven Reconfiguration of GRNs Is Centered on Noise and H3K9ac Changes

**Figure 7:**
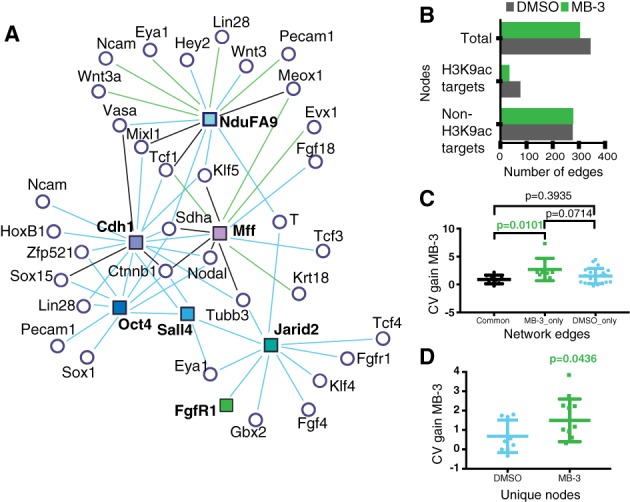
Network analysis of MB‐3‐treated cells shows destabilization following Kat2a inhibition. **(A):** Association network diagrams of Kat2a‐dependent H3K9ac target genes upon DMSO and MB‐3 treatment. Square nodes, Kat2a‐dependent H3K9ac target genes; white nodes, correlated genes; edge color, correlation observation in each condition (black = common; blue = DMSO only, green = MB‐3 only). **(B):** Enumeration of total network edges centered on Kat2a‐dependent H3K9ac target and nontarget genes upon treatment of TNGA embryonic stem (ES) cells in 2i with either DMSO or MB‐3. **(C):** Coefficient of variation (CV) gain [(CV_MB‐3_‐CV_DMSO_)/CV_DMSO_] for DMSO unique, MB‐3 unique, and common edges in the remodeled networks centered on Kat2a‐dependent H3K9ac targets (shown in A and B). **(D):** CV gain in network nodes exclusively present in DMSO or MB‐3 treatment of TNGA ES cells in 2i. All analyses used paired Student's *t* test. Abbreviation: DMSO, dimethyl sulfoxide.

We focused on control and MB‐3 treated cells in 2i culture conditions, where the transcriptional distance between states is more notable, and attempted to infer GRNs through a combination of binary and correlation methods we recently used in an adult differentiation system 35 (Supporting Information File S6). Given the proposed association from our data between loss of Kat2a‐dependent H3K9ac and gene expression variability, we asked if this phenomenon might be instrumental in reconfiguring GRNs. Reassuringly, the inferred control network was strongly nucleated in pluripotency factor *Oct4/Pou5f1* (Fig. 7A), as well as in Wnt signaling elements *Ctnnb* and *Cdh1*, which likely reflect GSK‐dependent Wnt activation in 2i culture conditions. Upon MB‐3 treatment, we observed a specific reduction of network connectivity amongst nodes dependent on Kat2a activity for H3K9ac (Fig. 7B). Pluripotency regulators *Pou5f1* and *Sall4*, as well as *Jarid2* showed complete loss of connectivity upon Kat2a inhibition, while the network around *Cdh1* was greatly reduced (Fig. 7A, 7B). Importantly, when focusing on remodeled edges built around the respiratory chain component *Ndufa9* and differentiation factor *Mff*, both H3K9ac differential targets, we observed that the new connections established upon MB‐3 treatment were with genes presenting significantly higher CV gains relative to non‐remodeled edges (Fig. 7C); as a trend, gains in CV were also higher relative to edges exclusively detected in DMSO‐treated cultures. A similar pattern of higher CV gains upon Kat2a inhibition was not observed in remodeled edges around nodes not strictly dependent on Kat2a for H3K9ac (Fig. 7D), suggesting a direct contribution to locus regulation. Furthermore, in interrogating the networks for genes exclusively connected upon Kat2a inhibition, we found that these presented higher CV gains than those genes exclusively present in the control network (Fig. 7E), suggesting a role for noise in network maintenance and reconfiguration.

The nature of the genes unique to each network underlies the phenotypes observed. In addition to *Pou5f1*, *Sall4*, and *Jarid2*, the control network uniquely also includes Fgf signaling elements, recently reported to be regulated by Kat2a 27, and *Id3*, a BMP target 21. Amongst the nodes specific to the MB‐3 network are endodermal (*Afp*), mesodermal (*Chrd*, *Wnt3a*) and NE (*Dmbx1*, *Evx1*) differentiation genes. Interestingly, the MB‐3 network also uniquely includes *Nanog*, whose presence in the network may reflect capture of the proposed early transition state into commitment (Fig. 5 and Supporting Information Fig. S3). Overall, our data support the notion that MB‐3‐mediated loss of H3K9ac at target loci critically reconfigures the architecture of the GRN sustaining pluripotency, through a direct effect on variability of gene expression.

## Discussion

We have shown that changes in the activity of the histone acetyl‐transferase Kat2a deplete a significant proportion of H3K9 acetylation sites at promoter locations and alter gene expression heterogeneity in mouse ES cells. Affected loci include key components of the pluripotency network, as well as general metabolic categories, which suggests that this mode of action may be extended to other cell types and developmental contexts. We have estimated heterogeneity on the basis of expression level CV and shown that Kat2a‐inhibited cultures exhibited higher CVs than controls, particularly when probed from naïve (2i) pluripotency conditions. Functionally, this translated into a curtailed ability to establish naïve pluripotency and an increased propensity to initiate ME differentiation, best explained by an increased probability to irreversibly exit the pluripotent state, without accompanying changes in cell cycle or apoptosis. Our recent report that loss of KAT2A can promote differentiation of human leukemic cells 20 is compatible with a generalization of the principles established in this work.

Notably, the radical changes observed upon MB‐3 treatment occur with minimal perturbation of average gene expression levels, sustaining the notion that variability of gene expression can, in itself, alter the probability of state transitions of individual cells. This is compatible with our previous observations in the hematopoietic system that lineage specification can be consequent to distinct individual molecular events, which are stochastic in nature and independently drive reorganization of transcriptional programs 9. Indeed, in mouse ES cells treated with MB‐3 and sampled from a newly established naïve pluripotency state, transcriptional heterogeneity resulted in a dramatic reconfiguration of GRNs, with destabilization of *Pou5f1/Oct4* connectivity, as well as of other transcriptional and signaling regulators of pluripotency, including transcriptional and cell–cell adhesion components of the Wnt pathway. Furthermore, GRN remodeling observed upon Kat2a inhibition included novel associations suggestive of differentiation, but also concomitant nucleation of the networks by *Nanog*, likely denoting the capture of an early and normally transient state in which differentiation programs are primed in the context of a subsisting pluripotency network. Although it was not based on known interactions, our experimental and modeling data lend support to this interpretation.

The underlying transcriptional variability that accompanies capture of the MN transition state is reminiscent of other transition events associated with enhanced heterogeneity 2, 3, 5, 7. Recently, it was suggested that heterogeneity of expression levels occurs primarily in genes which decrease their expression at fate decisions 36 but that this heterogeneity resolves on commitment to a stable, differentiated state, requiring a dynamic means of transcriptional regulation. In line with this view, Ahrends et al. 1 have modeled the contribution of transcriptional noise during commitment and progression and adipocyte differentiation and found that while low levels of noise ensure lineage commitment, noise must be limited for differentiation to progress. Lysine acetylation is a highly dynamic post‐translational modification, and selective perturbation of H3K9 acetylation constitutes an interesting regulatory paradigm, as it associates with maintenance, but not with initiation, of transcriptional activity 37. As such, reduction in H3K9 acetylation at specific loci is likely to destabilize, but not abolish, the transcriptional processes, and result in stochastic gene expression, on which other more stable epigenetic marks can act. At a molecular level, our results mirror the effects of the yeast Kat2a homolog Gcn5 on transcriptional noise 18, 34, and thus suggest that in eukaryotes, H3K9 acetylation may play a central role in the control of heterogeneity. Our observations also suggest that dynamic control of transcriptional noise may be key to efficient state transitions.

Early reports on Kat2a null mouse ES cells did not indicate a requirement for maintenance of pluripotent cultures 38. However, they did denote an acceleration of differentiation of embryoid bodies, and reduced contribution to chimaeras, both of which are compatible with our observed destabilization of pluripotency and anticipation of early ME differentiation, as well as with the proposition that persistent high noise levels prevent terminal execution of lineage programs. Indeed, Kat2a‐null embryoid bodies, particularly in a conditional knockout framework, constitute a good system in which to test the contribution of gene expression heterogeneity at different stages of differentiation progression, and in different lineages. Recent detailed analysis of the molecular pathways underlying the Kat2a null defects in embryoid bodies 27 identified an association with general metabolic pathways, which we also see in our work, and a specific defect in Fgf signaling. Although Fgf signaling is actively repressed in our experimental context, it is noteworthy that Fgfr1 is a direct target of Kat2a‐mediated H3K9 acetylation, and that Fgf4 and Fgfr1 exhibit reduced network connectivity and increased variability of gene expression upon MB‐3 treatment.

Finally, it should be noted that we conducted the molecular analyses using MB‐3 chemical inhibition of Kat2a at fairly high concentration which has known limitations 39; however, we were able to validate the cellular consequences of Kat2a inhibition with gene expression knockdown, suggesting that the observed results are biologically specific to Kat2a. We also used various mouse ES cell lines, including TNGA cells that allow for the direct exclusion of committed cells and the verification of increased culture heterogeneity by a simple measure. TNGA are heterozygous for Nanog, and consequently partially defective for the autoregulatory loop that maintains Nanog expression 16 and re‐enforces pluripotency. The loss or inhibition of Kat2a in these cells may be sufficient to affect the metastability of the primed pluripotency state and enhance heterogeneity. The reproducibility of cellular events in non‐TNGA cells suggests a more universal mechanism of noise enhancement, but this may need to be experimentally validated in other ES cell lines, and distinct cell types. Along the same line, it will be interesting to test whether other epigenetic regulators suggested to regulate transcriptional noise in yeast 18 have a similar impact on exit from pluripotency, or if sequence editing of locus‐specific regulatory elements targeted by H3K9 acetylation promotes exit from pluripotency in wild‐type mouse ES cells. The recent demonstration that locus‐specific editing of promoter acetylation alters the frequency of transcriptional bursting 40, which may in turn modify transcriptional noise 18, supports the feasibility of the proposed approaches. The on‐going expansion of CRISPR‐based tools for gene and epigenetic editing 41 should further assist in these experiments and provide an invaluable resource for dissection of cell state transitions in multiple stem cell systems and during development.

## Conclusion

In summary, we use Kat2a inhibition in mouse ES cells to establish a relationship between transcriptional heterogeneity and cell fate transitions, through manipulation of promoter acetylation and with destabilization of underlying transcriptional networks. This relationship may extend to other normal and malignant stem cell systems.

## Author Contributions

N.M.: conception and design, collection and assembly of data, data analysis and interpretation, manuscript writing, final approval of manuscript; S.E.: collection and assembly of data, data analysis and interpretation, final approval of manuscript; D.S.: data analysis and interpretation, final approval of manuscript; R.K.: data analysis and interpretation, final approval of manuscript; A.F.D.: collection and assembly of data, final approval of manuscript; T.B.: collection and assembly of data, final approval of manuscript; M.F.: supported data analysis and interpretation, final approval of manuscript; C.P.: conception and design, data analysis and interpretation, financial support, manuscript writing, final approval of manuscript.

## Disclosure of Potential Conflicts of Interest

The authors indicated no potential conflicts of interest.

## Supporting information


**Supplementary File 1**. ChIP‐seq peak coordinates for H3K9ac binding in DMSO and MB‐3‐treated TNGA ES cells.

**Supplementary File 2**. Nearest gene to DMSO exclusive H3K9ac peaks −1 to +0.5 kb of the transcriptional start site (TSS).

**Supplementary File 3**. Gene Ontology classification of DMSO exclusive H3K9ac TSS peaks (PANTHER vs.13.1 over‐representation test; Binomial test with Bonferroni correction, p < .05; fold change cut‐off 1.5)

**Supplementary File 4**. RNA‐seq differential gene expression analysis of TNGA ES cells treated with MB‐3 vs. DMSO. (Analysis performed using DESeq2; lfcSE: log2 fold change standard error, stat: test statistic, FDR: false discovery rate)

**Supplementary File 5**. Coefficient of variation (CV) calculations for individual genes analyzed by single‐cell qRT‐PCR in TNGA ES cells treated with DMSO and MB‐3 in SL and 2i culture conditions.

**Supplementary File 6**. Significant network associations (Spearman correlation and Odds Ratio) in TNGA ES cells treated with DMSO and MB‐3 in 2i conditions.

**Supplementary Figure 1**. MB‐3 treatment of mouse ES cells results in enhanced heterogeneity of gene expression from endogenous and reporter *Nanog* loci. A. Kat2a knockdown by shRNA in TNGA cells increases the mid Nanog‐GFP population proportionally to degree of knockdown. Lower panel shows the correlation between knockdown efficiency (Kat2a expression level assessed by RT‐qPCR) and heterogeneity of Nanog expression (Robust CV of Nanog‐GFP profiles) at Day 8 after transfection. Representative example from 2 biological replicates. **B.** Kat2a knockdown is also achieved in the destabilized reporter line, Nanog‐VNP, with equivalent linear relationship between knockdown efficiency (24 hours after transfection) and increase in Nanog‐VNP heterogeneity (Day 6). Representative example from 2 biological replicates. **C.** Destabilized Nanog reporter expression, Nanog‐VNP, following 1 day (left) or 2 days (right) MB‐3 treatment.

**Supplementary Figure 2**. MB‐3 treatment does not change apoptosis or cell cycle of mouse ES cells and has no effect on neuro‐ectodermal differentiation. A. TNGA cell fluorescence profile upon exposure to MB‐3 or DMSO in the presence of 2i medium, following routine culture in ESLIF. B. Quantification of apoptosis in TNGA cells in ESLIF supplemented with MB‐3 or DMSO for 1–3 days, or freshly transferred from ESLIF to 2i conditions and similarly treated for 1–3 days with MB‐3 or DMSO. Bar charts summarize average Annexin V+ proportions in high Nanog‐GFP and low Nanog‐GFP populations in 5 independent experiments (mean ± SEM; Student's *t*‐test, * p < .05). C. Representative cell cycle traces of TNGA cells treated with either MB‐3 or DMSO for 2 days in ESLIF conditions. D. After 3 passages in 2i medium, TNGA cells show no alteration of their fluorescence profile when cultured in the presence of MB‐3. E. Effects of exposure of *Sox1‐GFP* mouse ES cells to MB‐3 or DMSO prior to transfer to neuroectodermal differentiation‐promoting conditions (n = 3). No significant differences in GFP levels were detected between the 2 treatments (Paired *t*‐test p > .05).

**Supplementary Figure 3**. Dynamic cell state transitions following MB‐3 treatment. A. Representative flow cytometry plots of Nanog‐GFP levels after 1 day (left panels) or 2 days (right panels) of treatment with DMSO, two concentrations of MB‐3 or N2B27 before exposing to ESLIF (experimental schema in Fig. 3). **B.** A three Gaussian Mixture Model best fits the Nanog‐GFP virtual ensemble distribution. **C.** Sorting of the ESLIF MN population and subsequent culture in ESLIF conditions supplemented with either DMSO or MB‐3 shows similar dynamics to those seen with the reversibility experiment, namely a transition toward both HN and LN states in DMSO, indicative of a transient population, and movement primarily toward the LN state with MB‐3 treatment.

**Supplementary Figure 4**. Single‐cell RT‐qPCR analysis of pluripotency‐ and differentiation‐associated genes. A. Panel of genes selected for analysis at the single‐cell level includes those associated with the pluripotency network and differentiation lineages (upper left) and those associated with signaling pathways (bottom left). Of these, different proportions of genes have H3K9 and H3K14ac associated with their genetic sequences (right). **B.** Individual Squared Coefficient of Variation (CV2) value changes for each gene in 2i (top) and ESLIF (bottom). **C**. Frequency changes of genes based on treatment in ESLIF (left) or 2i (right) where each point represents a frequency of positive gene expression in either DMSO (lighter color) or MB‐3 (darker color) with a line between the two. Red lines represent significant expression frequency changes (Fischer test, p < .05). **D.** Violin‐plot representation of single‐cell RT‐qPCR average gene expression levels upon treatment with DMSO or MB‐3, in ESLIF (SL) and in 2i culture conditions. Differences between treatments are not significant (*t*‐test SL, p = .3498; 2i, p = .07501). **E.** t‐SNE plots of transcriptional profiles of individual TNGA cells treated with DMSO or MB‐3 in either ESLIF (left) or 2i culture conditions (right). Data as in Figure 6A in the main text, but with t‐SNE plots calculated separately for each culture condition.

Appendix S1: Supplementary Material
