# Supplementary material for: Histone Acetyltransferase KAT2A Stabilizes Pluripotency with Control of Transcriptional Heterogeneity
Source: Stem Cells. 2018 Oct 17;36(12):1828–38. doi: 10.1002/stem.2919 (PMC6334525; doi:10.1002/stem.2919)
Supplement: Supplementary file 11 — Appendix S1: Supplementary Material [file STEM-36-1828-s002.docx]

**SUPPLEMENTARY FILES**

**Supplementary File 1 (.xls). ChIP-seq peak coordinates for H3K9ac binding in DMSO and MB-3-treated TNGA ES cells.**

**Supplementary File 2 (.xls). Nearest gene to DMSO exclusive H3K9ac peaks -1 to +0.5kb of the transcriptional start site (TSS).**

**Supplementary File 3 (.xls). Gene Ontology classification of DMSO exclusive H3K9ac TSS peaks** (PANTHER vs.13.1 over-representation test; Binomial test with Bonferroni correction, p<0.05; fold change cut-off 1.5)

**Supplementary File 4 (.xls). RNA-seq differential gene expression analysis of TNGA ES cells treated with MB-3 vs. DMSO.** (Analysis performed using DESeq2; lfcSE: log2 fold change standard error, stat: test statistic, FDR: false discovery rate)

**Supplementary File 5 (.xls). Coefficient of variation (CV) calculations for individual genes analysed by single-cell qRT-PCR in TNGA ES cells treated with DMSO and MB-3 in SL and 2i culture conditions.**

**Supplementary File 6 (.xls). Significant network associations (Spearman correlation and Odds Ratio) in TNGA ES cells treated with DMSO and MB-3 in 2i conditions.**

**SUPPLEMENTARY TABLES**

**Supplementary Table 1. Primer sequences used for scRT-qPCR experiments.**

| Gene | Forward | Reverse |
| --- | --- | --- |
| Afp | TTCTCATTAACCTGGTGAAGCAA | TCTCCAAAAGGCCCGAGAAA |
| Axin2 | CTAGACTACGGCCATCAGGAA | GCTGGCAGACAGGACATACA |
| Cdh1 | CAATGCCTGCTCTTGATGGT | GGGAGATCTGACTGCCTCTG |
| Cdh2 | GGGATGAGACCACAAGATAGGA | AAACTCCCTTTATCTGCAACCA |
| Cdx2 | TCCTGCTGACTGCTTTCTGA | CCCTTCCTGATTTGTGGAGA |
| Chordin | GAGAAGGAGTGGTGCTGAGG | AGGAGTTCGCATGGATATGG |
| Ctnnb1 | GGTGGGCTGGTATCTCAGAA | CTTGTGATCCATTCGTGTGC |
| Dll1 | GACCATGGAGCCGAGAAGA | CCTGACCGTGGCTTCATCT |
| Dmbx1 | CCTGGGTGCAAATGACTGAT | TCAGGTGGCCTTGCTCTTTA |
| Dppa4 | TTGCTGCAAGTAGGTCCTGG | GCTGAACTGGTTATGACGCC |
| Emx1 | ACTTCTACCCCTGGGTGCTT | GCTCCCACCACGTAGTGATT |
| Eomes | AGCCAGCCCTACAACAAATG | CCAGCCCTACAACAAATGGT |
| Esrrb | GGGATCTCTTTCTCTCGTTCC | TCGTATTTCTCAAGCTCTGTCC |
| Evx1 | TACAAACCTTCAGCGCCTCT | AAGGACCACTTCCTCCCAGT |
| Eya1 | CCGTGTCTGGGATTCTTGTT | GTCTGGGTGGGAAGGAAAGA |
| FGF18 | GGCGATAGGATTCCACTGTT | TTTCTCGCAGTTTCCTCGTT |
| Fgf4 | GGCCACTCCACAGAGATAGG | ACTTGGGCTCAAGCAGTAGG |
| Fgfr1 | AGCTGTGGTTTGGGTCATTG | CCTAGCCCTCTTTGCCAAATG |
| Flk1 | TTTGCGTGCTCTTCACAGTC | AGCAAGCTGCATCATTTCCT |
| Gata4 | GCCCAGTTGTGCAGCTAATG | GGTGCAGATGAGCCATAGTC |
| Gbx2 | GCAGTCGGTTGATTTTGGAG | CGACATGGCTCAGATAGGAT |
| Gsc | GGAGAAGGTGGAGGTCTGGT | TGAGGACGTCTTGTTCCACTT |
| Hes1 | GCCAATTTGCCTTTCTCATC | AGCCACTGGAAGGTGACACT |
| Hes5 | CTTCTGCGAAGTTCCTGGTC | GAGGAAACACCTGCAGTTCC |
| Hey-2 | GTAGCTGCTCCTCCTTCGTC | CCAGGGTAATTGTTCTCGCT |
| HoxB1 | CAGAGGCTGGCTTACGAGAC | TTCTTGGTTGAGGCTTGCTT |
| Hoxb8 | GCGCTGTGAGCATTGTTAAA | TAACTCTTCCGCCCTTTTCA |
| HoxC5 | CATTTCCTCTCCAGCCTTCC | CCAGAGCCACTCTGTTTCCT |
| HoxC9 | AGAACCGGAGGATGAAGATG | TTTGTGTTTCCCTTGGCTGT |
| Id3 | CCAGCCCTCTTCACTTACCC | GGCCACCCAAGTTCAGTCC |
| Irx1 | TAAAGGACAAACGTGACGGC | CTTCCCAAGCGCTTAGGC |
| Jarid2 | CGTGGTAGTGGAGTAGCTGTTG | GCTCAGTGCAATGTGGGTAG |
| Kdm1a | CATGGTGCTCTGTTGAGTGG | ATGCCGTTGGATCTCTCTGT |
| Krt18 | CCAGCAACTCCATGCAAAC | CAGGGGTTCCCTCCTTCTCT |
| Klf4 | CCAGCAAGTCAGCTTGTGAA | TTTGTAAGTCCGGGCATGTT |
| Klf5 | ACTACAGGAAGCACACGGG | TCGCTCGCTCAGTTCTGG |
| Krox20 | AATGGCTTGGGACTGACTTG | TGAGATGGCCAGAGAAACCT |
| Lef1 | GCGAATGTCGTAGCTGAGTG | GCTGTCTCTCTTTCCGTGCT |
| Lin28 | CCAGGAACCTCATTCTTTCC | ACCCGAGGCTCTCTATCTCC |
| Mbd3 | GGAAACACACCTGGCTATGC | GAAAGTGACTTCCTGGTGGG |
| Meox1 | GCACAAGAGCTGATGGATGA | ACGCAGGATAGGTCCAAATG |
| Mff | TAACAGCTGGCTCTGGTTTC | CACCACAGGCCTTCACTCTAC |
| Mixl1 | GGCAGCTTCCAGTTAACCAA | CTGAGTCCCAACCAGAAAGG |
| Myc | CACCGCCTACATCCTGTCC | TCAACTGTTCTCGTCGTTTCC |
| Nanog | CTTTCACCTATTAAGGTGCTTGC | TGGCATCGGTTCATCATGGTAC |
| Ncam | CGGTGTGTCTTATGTCTATGTGG | TGAGGACATAGAATCCTGTTGG |
| NduFA9 | GATCCAGATGCCGTAGGAAA | AAATGGACTCAGCCCAAAGA |
| Nestin | AGCAAGTGAATGGGAGGATG | GCCTAGATGCACAGGAGACC |
| Neurog1 | AAGCTGCCCTCGGTCTATTT | GGGTCAGTTCTGAGCCAGTC |
| Neurog2 | GCAACTGGTCCCTGTGATCT | ACAGGTGAAATTCCCACAGC |
| Nkx2.5 | TCGACGGATTCCACACTAGG | AGCGCACTCACTTTAATGGG |
| Nodal | AGCCACTGTCCAGTTCTCCAG | GTGTCTGCCAAGCATACATCTC |
| Notch1 | TCCAATGTGCATTGTGGACT | TGCAAGAATCTGCTGTGAGC |
| Notch2 | CTGACTTATGCGATGGTGGG | ATGCAAGACTTCAAGTGGCC |
| Notch4 | GGGTCTTCCAGTTCACCAAG | TCAACCGGACATCCTAAACC |
| Noxa | CACCTTAAATCCAGCTGTCCC | ACCCAGAAGTACAGATGTCCC |
| Nr6a1 | AGAAGCCTAGACCCTTTGCC | CCACAGCTTCCATTTGAGCC |
| Oct4 | ACCACACTCTACTCAGTCCC | TGGTGCCTCAGTTTGAATGC |
| Otx1 | CTGCCCTTGATCTAGCTCCC | AAGCTTGAATCAGTCCAGCG |
| Otx2 | CTGGGCTGAACATTCCAGTT | GTCCATTTCAGGTTGCTGGT |
| Pax3 | AGTCACATTGATTTGGAGGCC | AGTATTGCACGCTGAGAAGC |
| P-cadherin | TCGATTCAAGAAACTGGCGG | CTTGAGATGCTGCTGTGACC |
| PDGFRa | CCACACTCAGCAGGGATACA | GGGCAAGCTTTGATTGTCAT |
| Pecam1 | TGCCTTGTTCATGTTGGGTA | TCTCCTGGAACCTCCTTTCA |
| Pou3f1 | CTCTCCTCCTTCAAGAACGC | CTCTGCTAGGCCAGGAACC |
| Ppia | TTACCCATCAAACCATTCCTTCTG | AACCCAAAGAACTTCAGTGAGAGC |
| PTK7 | GGC CTC AGT CTC CTC TCC TC | CTC CCT CCC TAC TTC CTC CA |
| Rai1 | CAGCTCACTCGGCTCATAGG | CACAGAAGCGAGCATGTTCC |
| Rara | CTGGATCTCGAGCTGAAGGG | ACTGAGGAGCAAAGACTGGG |
| Rex1 | GCGGTGTGTACTGTGGTGTC | GACAAGCATGTGCTTCCTCA |
| Sall4 | CTCCCACAAACCACCTTCC | CGGGCACTGAAGACAACC |
| Sdha | CCGCTCCTACTGATGAAACC | AAGTCTGGCGCAACTCAATC |
| Snai1 | ACCCACTCGGATGTGAAGAG | AGCCAGACTCTTGGTGCTTG |
| Sox1 | AGACAGCGTGCCTTTGATTT | TGGGATAAGACCTGGGTGAG |
| Sox15 | AGCAGAGGGTAGAAAGAGCG | CTTCCAGAGCGCTTGAATCC |
| Sox17 | TTCTGTACACTTTAATGAGGCTGTTC | TTGTGGGAAGTGGGATCAAG |
| Sox2 | CATGAGAGCAAGTACTGGCAAG | CCAACGATATCAACCTGCATGG |
| SPRY4 | ATGGTGGATGTCGATCCTGT | GGAGGGGGAGCTACAGAGAC |
| T | CTGGGAGCTCAGTTCTTTCG | GTCCACGAGGCTATGAGGAG |
| Tcf1 | TGCAAGAGGAGGGACTAGGA | TGGGAAGAGCCTGATAGGAA |
| Tcf15 | CACTCCTGCGTTGTGTAAGG | CTGGATGGCTAGATGGGTCC |
| Tcf3 | ACGGTTCTGGATGAGACAGG | AAGCAGGGAGCTGTTCAGTG |
| Tcf4 | TTTCAATGTGTGGCTGAGTG | GTGGGCCTCTTGAAGGAAGT |
| Tubb3 | GGCGCATGTCTATGAAGGAG | GGCTACCTTGACGTTGTTGG |
| Vasa | GTGCCTCCCAGCTTCAGTAG | AACTGGATTGGGAGCTTGTG |
| Wnt1 | TCACTGCTCCTCTGTTCTGC | GAAATACTGATCCGGTGGGC |
| Wnt3 | CTAATGCTGGCTTGACGAGG | ACATGGTAGAGAGTGCAGGC |
| Wnt3a | CATACAGGAGTGTGCCTGGA | AATCCAGTGGTGGGTGGATA |
| Wnt8b | CGGCTTGTCATTTCTTCCAT | GTAGTGCATGGGCAGGAAGT |
| Zfp281 | TGGCTCTGCTCTAAACCACTT | GAGTTCATAAGACTAAACACCACCA |
| Zfp521 | GCGTCGTTCCAAAGACAAAG | GGAGATGAAGCCATTTGGAG |
| Zic2 | AACCCATAGCACACATCACC | GCACTGAAACATCACATGTAAACC |

**Supplementary Table 2. Taqman assays used for scRT-qPCR experiments.**

| Gene | Reference |
| --- | --- |
| Hprt1 | Mm01545399.m1 |
| Atp5a1 | Mm00431960.m1 |
| Als2 | Mm01255664.m1 |
| Bbs1 | Mm01239251.m1 |
| Ccdc57 | Mm01253284.m1 |
| Cisd3 | Mm01298885.g1 |
| Crem | Mm01230944.g1 |
| Ddit4 | Mm00513313.m1 |
| Dsn1 | Mm00482243.m1 |
| Dusp1 | Mm00457274.g1 |
| Etnk2 | Mm00615296.m1 |
| Fadd | Mm00438861.m1 |
| Hnrnph3 | Mm01032120.g1 |
| Klf4 | Mm00516104.m1 |
| Lrrc57 | Mm00712359.m1 |
| Med18 | Mm00509065.m1 |
| Pink1 | Mm00550827.m1 |
| Poli | Mm01262544.m1 |
| Rfesd | Mm00841715.m1 |
| Scfd2 | Mm00724490.m1 |
| Tmem25 | Mm00503605.m1 |
| Tsn | Mm00457052.m1 |
| Usp2 | Mm00497452.m1 |
| Zfp438 | Mm00724584.m1 |
| Atm | Mm01177457.m1 |
| Brwd1 | Mm01209842.m1 |
| Gpr56 | Mm00817704.m1 |
| Hlf | Mm00723157.m1 |
| Hspa5 | Mm00517690.g1 |
| Jmjd5 (Kdm8) | Mm00513079.m1 |
| Mbd3 | Mm00488961.m1 |
| Meis2 | Mm00487748.m1 |
| Mrps24 | Mm00466026.m1 |
| Myo5C | Mm00520289.m1 |
| Nat10 | Mm00462302.m1 |
| Ncor1 | Mm01333102.m1 |
| Nfe2L1 | Mm00599712.m1 |
| Rai1 | Mm01163529.m1 |
| Rad17 | Mm01288359.m1 |
| Rara | Mm01296312.m1 |
| Rel | Mm01239661.m1 |
| Rpl23 | Mm00787512.s1 |
| Rps6Ka5 | Mm00463868.m1 |
| Smad4 | Mm03023996.m1 |
| Sox17 | Mm00488363.m1 |
| Spry4 | Mm00442345.m1 |
| Top1 | Mm00493749.m1 |
| Ubc | Mm02525934.g1 |

**SUPPLEMENTARY EXPERIMENTAL PROCEDURES**

**Microscopy**

Immunofluorescence microscopy was performed as described [[1](#_ENREF_1)] using a Nanog antibody (14-5761-80, eBioscience) on a Zeiss Axiovert confocal microscope. For quantification of confocal images, nuclei centers were manually selected and the mean fluorescence/cell quantified by average greyscale value using a 16x16 pixel square area around each seed-point with an in-house Python script. Data was analysed using R.

**RNA-sequencing**

RNA was extracted using Trizol reagent (ThermoFisher) as per manufacturer’s instructions, and libraries prepared using PolyA enrichment. All libraries were single-end sequenced on an Illumina HiSeq 4000 instrument. For RNA-seq analysis, trimmed reads were aligned to the mouse mm10 genome using Bowtie for Illumina [[2](#_ENREF_2), [3](#_ENREF_3)] and quantified at exonic regions using the GenomicAlignments Bioconductor package in R [[4](#_ENREF_4)]. Statistical comparison of the DMSO versus the Mb3 treated samples was performed using the DESeq2 package [[5](#_ENREF_5)] with an adjusted p-value cut-off of 0.05. Gene set enrichment analysis was performed using the GSEA tool [[6](#_ENREF_6)] with the RNA-seq gene expression fold changes against a published gene set of 474 Kat2a direct targets [[7](#_ENREF_7)].

**ChIP-sequencing**

Chromatin Immunoprecipitation was performed as published [[8](#_ENREF_8)] using an antibody against H3K9ac (ab4441, AbCam). ChIP sequencing experiments were performed in biological duplicates and data were aligned to the mm9 genome with BWA before merging replicates together with samtools merge [[9](#_ENREF_9)] due to the relatively low number of reads. Top enriched genomic regions compared to the paired input (peaks) were identified from merged samples using MACS2 [[10](#_ENREF_10)]. All peaks with q-value < 10-2 were retained. Gene identities were obtained using Genomic Regions Enrichment of Annotations Tool (GREAT, version 3.0.0; [[11](#_ENREF_11)]) to assign gene identities to the H3K9ac peaks obtained from the DMSO and MB-3-treated cells. Genomic regions for gene identification was restricted to 1kb upstream and 500bp downstream of the TSS, to infer genes regulated at the promoter level. These identities were used to obtain targets unique to DMSO treatment. For these, we used the ENCODE ChIP-Seq Significance Tool [[12](#_ENREF_12)] to obtain putative regulatory transcription factors. Functional classification was performed using the over-representation test for ‘slim’ Gene Ontologies with Binomial test and Bonferroni correction in PANTHER [[13](#_ENREF_13)] vs.13.1.

**Quantitative PCR**

Bulk quantitative RT-PCR was performed following RNA capture by Trizol (ThermoFisher) and reverse transcription by Superscript III RT First Stand Synthesis (ThermoFisher). Quantification was performed with Quantifast SYBR Green (Qiagen), before running on a Rotor-Gene Q (Qiagen) real-time PCR cycler, using primers in Supplementary Table 5-6. Initial sample concentrations were calculated using an in-house MAK2 method.

**Short Hairpin RNA**

Knockdown of Kat2a was performed using short hairpin RNA targeted against Kat2a using the mCherry reporter system from VectorBuilder (Cyagen), using pLV[shRNA]-mCherry-U6>Scramble_shRNA (VB161031-1099uhp; CCTAAGGTTAAGTCGCCCTCG), pLV[shRNA]-mCherry-U6>(Kat2ashRNA) (VB161031-1094sqf; GCTACCTACAAAGTCAATTA), pLV[shRNA]-mCherry-U6>(Kat2ashRNA2) (VB161031-1096tdc; GAGATCATCAAGAAGTTGAT) and pLV[shRNA]-mCherry-U6>(Kat2ashRNA3) (VB161031-1097hsq; GAAGCCTTCTACGGTCCATT). Lentiviral packaging and transduction was performed according to published protocols [[14](#_ENREF_14)].

## Kinetic modelling of Reversibility

## Defining Nanog transcriptional states

The distribution of the Nanog-GFP fluorescence after exposing TNGA cells to 1 or 2 days of either serum + LIF medium supplemented with DMSO or MB-3, or 1-2 days in N2B27 basal differentiation medium was quantified by flow cytometry immediately after treatment and for 1-3 days after the cells had been replated into serum + LIF conditions. The fluorescence distributions from each of these samples (32 in all) were randomly sampled to select *2,290* cells from each condition and combined to an ensemble virtual hybrid histogram and fitted using a Gaussian Mixture Model. This procedure was repeated 1,000 times to make sure there was no bias in sampling the cells from the different conditions. To this virtual hybrid distribution, between *1* and *4* component Gaussians mixture distributions were fitted using the MATLAB function *fitgmdist* [[15](#_ENREF_15)]. The selection of the best fitting was based on the Akaike information criterion (AIC), which includes the negative log likelihood of the fitting and the number of components of the model added as a penalty (the smallest the AIC is the better the fitting is). Following the AIC, *3* Gaussians were found to best fit the dataset.

## Clustering the cells in each condition to the three Nanog subpopulations

The clustering method was based on a soft clustering approach: each cell has a given probability of allocation to any one of the three Gaussians according to the probability density function (PDF) of each of the three components (N(μ_1_, σ^2^_1_), N(μ_2_, σ^2^_2_), N(μ_3_, σ^2^_3_), where μ and σ^2^ donate to the mean and variance respectively of a normal distribution) and their proportion. The cell’s PDF of each component is multiplied by the component proportion, creating 3 weighted PDFs for each cell, normalized by the sum of the 3 PDFs to get probabilities of assigning the cell to the different states.

Using these probabilities, each cell was assigned to one of the three clusters via a random process that was repeated 100 times, which is conclude to an average proportion of cells in each population, alongside confidence interval estimates.

Modelling the transition kinetics between the three Nanog states

A simple model of cells changing their Nanog state amongst HN, MN and LN states was fitted to the clustering output using the following three differential equations:

$$HN\begin{matrix} A1 \\ \rightleftarrows\\ A2 \end{matrix}MN\begin{matrix} A3 \\ \rightleftarrows\\ A4 \end{matrix}LN$$

$$\left( 2 \right) \frac{dHN}{dt}=A_{2}\cdot\left[ MN \right]-A_{1}\cdot\left[ HN \right]$$

$$\left( 3 \right) \frac{dMN}{dt}=A_{1}\cdot\left[ HN \right]+A_{4}\cdot\left[ LN \right]-\left( A_{2}+A_{3} \right)\cdot\left[ MN \right]$$

$$\left( 4 \right) \frac{dLN}{dt}=A_{3}\cdot\left[ MN \right]-A_{4}\cdot\left[ LN \right]$$

A_1_, A_2_, A_3_ and A_4_ in Eq. (2-4) are rate constants with dimension of$\frac{1}{day}$, representing cell movement forward and backward from HN to MN and MN to LN respectively. Estimation of the rate constants was done in an iterative unconstrained nonlinear optimization using the Nelder-Mead simplex algorithm as described in [[16](#_ENREF_16)].

**Single-cell Transcriptional Analysis**

Single cell transcriptional analysis was performed using the Fluidigm Biomark technology. Subsequent quality control was performed, including rejection of genes with aberrant melting curves and rejection of cells without standard expression of the housekeeping control or with fewer than 10% of genes expressed. All analysis was performed using R software, and Ct values were normalized to housekeeping controls (*Atp5a1* and/or *Ppia*) before linearization. Differential gene expression analysis used the nonparametric Mann Whitney U test with a Bonferroni adjusted p-value of 0.05 divided by the number of tests, on continuous expression data. Differences in frequency of expression were assessed with Fishers Exact test, also with a Bonferroni corrected p-value. For dimensionality reduction, the data was scaled in order to utilize the full dataset, namely by log transforming the linearized expression values and translating such that the lowest expression value was 0. t-distributed Stochastic Neighbour Embedding (tSNE) was performed using the Rtsne package [[17](#_ENREF_17)] with parameters of theta=0, max_iter=2000 and perplexity=25. Squared Coefficient of Variation (CV2) estimates for each gene (not including missing values) were bootstrapped and significance tested using the Mann Whitney U test with a Bonferroni corrected p-value; differences in ensemble CV values were tested using the Paired Student’s t-test. For correlation analysis, continuous (levels of expression) and binary (expressed vs. non-expressed) data were handled separately and combined after analysis, as published [[14](#_ENREF_14)]. Briefly, co-expressed continuous gene expression data was correlated using pairwise Spearman’s correlation and those with p < 0.001 with an absolute correlation above 0.6 were retained and plotted. Binarised data, excluding genes for which the proportion of variable cells was <10%, was subjected to an Odds Ratio test and those with an absolute correlation above 0.8 were retained and plotted together with the Spearman correlations. Networks were drawn using the qgraph package in R.

**SUPPLEMENTARY REFERENCES**

1. Kalmar T, Lim C, Hayward P, et al., Regulated fluctuations in nanog expression mediate cell fate decisions in embryonic stem cells. PLoS Biol, 2009. 7(7): p. e1000149.

2. Huber W, Carey VJ, Gentleman R, et al., Orchestrating high-throughput genomic analysis with Bioconductor. Nat Methods, 2015. 12(2): p. 115-21.

3. Langmead B, Trapnell C, Pop M, et al., Ultrafast and memory-efficient alignment of short DNA sequences to the human genome. Genome Biol, 2009. 10(3): p. R25.

4. Lawrence M, Huber W, Pages H, et al., Software for computing and annotating genomic ranges. PLoS Comput Biol, 2013. 9(8): p. e1003118.

5. Love MI, Huber W, and Anders S, Moderated estimation of fold change and dispersion for RNA-seq data with DESeq2. Genome Biol, 2014. 15(12): p. 550.

6. Subramanian A, Tamayo P, Mootha VK, et al., Gene set enrichment analysis: a knowledge-based approach for interpreting genome-wide expression profiles. Proc Natl Acad Sci U S A, 2005. 102(43): p. 15545-50.

7. Hirsch CL, Coban Akdemir Z, Wang L, et al., Myc and SAGA rewire an alternative splicing network during early somatic cell reprogramming. Genes Dev, 2015. 29(8): p. 803-16.

8. Tzelepis K, Koike-Yusa H, De Braekeleer E, et al., A CRISPR Dropout Screen Identifies Genetic Vulnerabilities and Therapeutic Targets in Acute Myeloid Leukemia. Cell Rep, 2016. 17(4): p. 1193-1205.

9. Li H, Handsaker B, Wysoker A, et al., The Sequence Alignment/Map format and SAMtools. Bioinformatics, 2009. 25(16): p. 2078-9.

10. Zhang Y, Liu T, Meyer CA, et al., Model-based analysis of ChIP-Seq (MACS). Genome Biol, 2008. 9(9): p. R137.

11. McLean CY, Bristor D, Hiller M, et al., GREAT improves functional interpretation of cis-regulatory regions. Nat Biotechnol, 2010. 28(5): p. 495-501.

12. Auerbach RK, Chen B, and Butte AJ, Relating genes to function: identifying enriched transcription factors using the ENCODE ChIP-Seq significance tool. Bioinformatics, 2013. 29(15): p. 1922-4.

13. Mi H, Muruganujan A, Casagrande JT, et al., Large-scale gene function analysis with the PANTHER classification system. Nat Protoc, 2013. 8(8): p. 1551-66.

14. Pina C, Teles J, Fugazza C, et al., Single-Cell Network Analysis Identifies DDIT3 as a Nodal Lineage Regulator in Hematopoiesis. Cell Rep, 2015. 11(10): p. 1503-10.

15. McLachlan GJ and Peel D, Finite mixture models. 2000, New York ; Chichester: Wiley.

16. Lagarias JC, Reeds JA, Wright MH, et al., Convergence properties of the Nelder-Mead simplex method in low dimensions. Siam Journal on Optimization, 1998. 9(1): p. 112-147.

17. van der Maaten L and Hinton G, Visualizing Data using t-SNE. Journal of Machine Learning Research, 2008. 9: p. 2579-2605.

**SUPPLEMENTARY FIGURE LEGENDS**

**Supplementary Figure 1. MB-3 treatment of mouse ES cells results in enhanced heterogeneity of gene expression from endogenous and reporter *Nanog* loci. A.** Kat2a knockdown by shRNA in TNGA cells increases the mid Nanog-GFP population proportionally to degree of knockdown. Lower panel shows the correlation between knockdown efficiency (Kat2a expression level assessed by RT-qPCR) and heterogeneity of Nanog expression (Robust CV of Nanog-GFP profiles) at Day 8 after transfection. Representative example from 2 biological replicates. **B.**  Kat2a knockdown is also achieved in the destabilised reporter line, Nanog-VNP, with equivalent linear relationship between knockdown efficiency (24 hours after transfection) and increase in Nanog-VNP heterogeneity (Day 6). Representative example from 2 biological replicates. **C.** Destabilised Nanog reporter expression, Nanog-VNP, following 1 day (left) or 2 days (right) MB-3 treatment.

**Supplementary Figure 2. MB-3 treatment does not change apoptosis or cell cycle of mouse ES cells and has no effect on neuro-ectodermal differentiation.** **A.** TNGA cell fluorescence profile upon exposure to MB-3 or DMSO in the presence of 2i medium, following routine culture in ESLIF. **B.** Quantification of apoptosis in TNGA cells in ESLIF supplemented with MB-3 or DMSO for 1-3 days, or freshly transferred from ESLIF to 2i conditions and similarly treated for 1-3 days with MB-3 or DMSO. Bar charts summarize average Annexin V+ proportions in high Nanog-GFP and low Nanog-GFP populations in 5 independent experiments (mean ±SEM; Student’s t-test, * p < 0.05). **C**. Representative cell cycle traces of TNGA cells treated with either MB-3 or DMSO for 2 days in ESLIF conditions. **D.** After 3 passages in 2i medium, TNGA cells show no alteration of their fluorescence profile when cultured in the presence of MB-3. **E.** Effects of exposure of *Sox1-GFP* mouse ES cells to MB-3 or DMSO prior to transfer to neuroectodermal differentiation-promoting conditions (n=3). No significant differences in GFP levels were detected between the 2 treatments (Paired t-test p > 0.05).

**Supplementary Figure 3. Dynamic cell state transitions following MB-3 treatment. A.** Representative flow cytometry plots of Nanog-GFP levels after 1 day (left panels) or 2 days (right panels) of treatment with DMSO, two concentrations of MB-3 or N2B27 before exposing to ESLIF (experimental schema in Figure 3). **B.** A three Gaussian Mixture Model best fits the Nanog-GFP virtual ensemble distribution. **C.** Sorting of the ESLIF MN population and subsequent culture in ESLIF conditions supplemented with either DMSO or MB-3 shows similar dynamics to those seen with the reversibility experiment, namely a transition towards both HN and LN states in DMSO, indicative of a transient population, and movement primarily towards the LN state with MB-3 treatment.

**Supplementary Figure 4. Single-cell RT-qPCR analysis of pluripotency- and differentiation-associated genes. A.** Panel of genes selected for analysis at the single-cell level includes those associated with the pluripotency network and differentiation lineages (upper left) and those associated with signalling pathways (bottom left). Of these, different proportions of genes have H3K9 and H3K14ac associated with their genetic sequences (right). **B.** Individual Squared Coefficient of Variation (CV2) value changes for each gene in 2i (top) and ESLIF (bottom). **C**. Frequency changes of genes based on treatment in ESLIF (left) or 2i (right) where each point represents a frequency of positive gene expression in either DMSO (lighter color) or MB-3 (darker color) with a line between the two. Red lines represent significant expression frequency changes (Fischer test, p < 0.05). **D.** Violin-plot representation of single-cell RT-qPCR average gene expression levels upon treatment with DMSO or MB-3, in ESLIF (SL) and in 2i culture conditions. Differences between treatments are not significant (t-test SL, p = 0.3498; 2i, p = 0.07501). **E.** t-SNE plots of transcriptional profiles of individual TNGA cells treated with DMSO or MB-3 in either ESLIF (left) or 2i culture conditions (right). Data as in Fig. 6A in the main text, but with t-SNE plots calculated separately for each culture condition.
